# Honeymoon, medical treatment or big business? An analysis of the meanings of the term “reproductive tourism” in German and Israeli public media discourses

**DOI:** 10.1186/1747-5341-8-9

**Published:** 2013-08-20

**Authors:** Sharon Bassan, Merle A Michaelsen

**Affiliations:** 1 Faculty of Law, Tel Aviv University, Ramat Aviv, PB 39040, Tel Aviv 69978, Israel; 2 Department of Medical Ethics and History of Medicine, University Medical Center Göttingen, Humboldtallee 36, Göttingen 37073, Germany

**Keywords:** Reproductive tourism, Metaphor, Medical tourism, Israel, Germany, Reproductive treatment, Infertility, Legal restrictions

## Abstract

**Background/Introduction:**

Infertile couples that travel to another country for reproductive treatment do not refer to themselves as “reproductive tourists”. They might even be offended by this term. “Tourism” is a metaphor with hidden connotations. We will analyze these connotations in public media discourses on “reproductive tourism” in Israel and Germany. We chose to focus on these two countries since legal, ethical and religious restrictions give couples a similar motivation to travel for reproductive care, while the cultural backgrounds and conceptions of reproduction are different.

**Results:**

Our research shows that the use of the metaphor “reproductive treatment” and its hidden messages depends on the writers’ intention and the target population. Although the phenomenon of patients travelling for reproductive treatment can fit into the definitions of tourism, the term emphasizes aspects that do not reflect patients’ reality. In both the German and the Israeli public media debate the term “reproductive tourism” is either used to criticize the economic aspects of the phenomenon or to attract patients as potential clients.

**Conclusions:**

Ethicists should be cautious when borrowing metaphors like “reproductive tourism” from the public debate. Our findings support Penning’s suggestion to use instead an unloaded term like cross-border reproductive care to describe the phenomenon in a more neutral way and to make it explicit whenever criticism is necessary.

## Background

In a study about so-called reproductive tourists the anthropologist Marcia Inhorn shows that many of the infertile couples going abroad for medical treatment do not regard themselves as tourists but instead as patients. They even feel offended by being called tourists because tourism is associated with fun, leisure or holidays, while they often experience their journey as highly stressful [1]. The term “reproductive tourism” is often used uncritically for a variety of practices. The article will describe the linguistic use and the dominant problems arising from it.

After introducing the basic facts of cross-border reproductive care and the general conditions that lead to the use of these procedures, we elaborate on the different legal-cultural backgrounds that motivate people from Israel and Germany to travel to other countries for reproductive treatment. We chose to focus on these two countries since legal, ethical and religious restrictions give couples a similar motivation to travel for reproductive care, while the cultural backgrounds and conceptions of reproduction are different.

Our aim is to examine if and how, by using the term “reproductive tourism”, the public media discourse in Germany and Israel implicitly conveys certain value judgements. We will address reasons, if existing, for using a loaded term rather than a more neutral one, such as “cross-border reproductive care”. By “neutral” we mean terms that are not morally loaded. The results of studies in the two countries will be compared, and hidden cultural, legal and social perceptions of the term “tourism” will be made explicit.

In light of the importance of the more general phenomenon of “tourism” in an era of globalization, our results contribute to a better understanding of the use of the term “reproductive tourism” and when and why it should be avoided.

### The phenomenon behind the term

The terms “medical tourism” and “health tourism” are used to describe treatment or surgery which has been planned in advance to take place outside a patient’s usual place of residence [2]. “Reproductive tourism” refers to infertile couples or individuals from one country purchasing reproductive or medical services from suppliers of such services in other countries, in order to conceive and have a child [3]. The term “reproductive tourism” covers various procedures which patients undergo such as *in-vitro* fertilization (IVF), egg and sperm donations, surrogacy, sex selection, and “pre-implantation genetic diagnosis” (PGD), whereas in other countries, the term also includes international adoption [4-6]. Other terms like “fertility tourism”, “procreative tourism” or “fertile tourism” all refer to the same practices.

According to the media [6,7] and to professional journals [8], “medical tourism” and “reproductive tourism” have recently become a growing international industry due to cheap and fast means of travel, the availability of services, the amount of information on the internet, and the possibility to share medical practices [2].

So far there has not been any official registration or standardization of the collection of data of cross-border reproductive treatment. Therefore, only incomplete data are available. In 2009 about 3 – 5% of all medical treatments in the USA were carried out on non-U.S.-citizens, for example from Canada, Europe or Asia. In Europe approximately 7 - 10% of medical care is provided to foreign nationals, mainly from other European countries [9]. Shenfield et al. estimate that in Europe 11 000 – 14 000 patients are travelling for reproductive treatment annually, based on a survey conducted in 2008/2009 [10]. The numbers of transactions in India, for example, show the explosive growth of this phenomenon: As Priti Sehgal states, in India “surrogacy is already an almost US$445 million business”, with a 150 percent rise in surrogacy cases during the last years [11].

### Reasons to travel

Patients choose medical (and, as part of it reproductive) treatments abroad for different reasons: to avoid rationing, such as waiting periods in their countries of origin [12]; to benefit from the low costs or higher quality of services overseas; to secure their privacy [13], or to have access to services not provided in their home country. When national laws in affluent countries restrict or prohibit medical forms of procreation or citizens’ access to fertility treatments, infertile individuals may privately exercise what they perceive to be their reproductive rights in other countries that have less restrictive laws regulating fertility transactions [14-16]. Such transactions mainly take place in the private market that is governed by the global economy, where the rules are mainly determined by liberal market values.

Countries that offer reproductive treatment not available in other countries therefore serve as a common destination for cross-border reproductive treatment. Today patients mainly travel from industrialized countries to less-developed nations when seeking affordable, high-quality treatment. For example, laws in the U.S. and UK do not allow a surrogate woman to charge the childless couple; therefore a smaller number of women tend to become surrogate mothers. In India there are no laws to prevent women from accepting compensation for surrogacy. Accordingly, it is easier for couples planning to have a baby to locate a surrogate mother in India than in the UK. In this light, there is a rising concern that women from vulnerable groups in target countries who often serve as surrogates or egg donors lack legal protection [17].

### The legal and cultural background in Germany and Israel

The recent legislation on reproductive treatments in both Germany and Israel may foster patients travelling to obtain reproductive treatments elsewhere.

In Germany the Embryonenschutzgesetz (Embryo Protection Law, ESchG) of 1991 was the first legislation to regulate artificial reproductive technologies. During the preparation of this law, the German lawyer and former president of the Federal Constitutional Court, Ernst Benda, called reproductive treatment that involves “unnatural splitting of motherhood” a threat to human dignity [18]. The inviolability of human dignity is established in the first paragraph of the German constitution. The fact that Benda referred to human dignity underlines the political and social importance of this issue in Germany.

Surrogacy and egg donation are forbidden by law in Germany. Other treatments, like IVF treatment only became available for people in a heterosexual marital or marital-like relationship; homosexual couples still are excluded from reproductive treatment.

Therefore, homosexual couples and infertile couples who choose egg donation or surrogacy have no other option but to look for such services elsewhere. Germans, for example, often travel to Belgium or Spain where more liberal legislation allows for egg donation and where homosexual couples also have access to reproductive treatment [19]. In German public media, the magazine “Der Spiegel” describes the role of the law as creating a global market for reproductive treatment: “Eventually, globalization brings dozens of new businesses into the country. For some time, strict laws in Europe and elsewhere have led to a flourishing of fertility tourism. The weak dollar has caused a veritable baby boom: some U.S. agencies are doing more than 40 percent of their business with customers from overseas^a^” [20].

In contrast, in Israel a more liberal choice policy is ensured by legislation. Parenthood has a broad social value and is promoted by a range of different cultures within the country, most notably Jewish culture and its reliance on religious scripture^b^. In addition, the Holocaust and the recent history of the state of Israel have created collective a pro-natalist awareness [21]. The trauma of the Holocaust and a communitarian sentiment to increase the number of Jewish people in Israel have been recognized as contributory as motivation for the liberal regulation of artificial reproductive technologies in the country [22]. This attitude towards reproduction in Israel may be important in explaining why travel to receive reproductive treatment is regarded as a means to a justifiable end – parenthood.

In Israel, every citizen is entitled to “infertility diagnosis and therapy”. Artificial fertilization “for the purpose of bearing a first and second child - for couples who do not have children from their current marriage, and also for a childless woman who wishes to establish a single parent family”, as specified in an addendum to the law, is part of the basic health package of medical services and medicines^c^. Historically, the Clalit health fund had no limitations on the number of treatments for the insured.

Most procedures conducted beyond the country’s borders are egg donation and surrogacy. Until recently (June 2010), when a new egg donation law was passed, it was not legally possible to receive an egg donation from a woman who was undergoing the process for the sole purpose of donating her eggs. Only women who underwent IVF treatment for themselves were allowed to donate eggs, but most of these women chose to freeze their eggs for later and avoid repeating the complicated procedure of egg retrieval. Therefore, in Israel the demand for eggs exceeded the supply.

With the new egg donation law introduced in June 2010, it is now possible to recruit egg donation from women who are not undergoing the procedure for their own purpose, but rather in exchange for compensation [23]. For now, private clinics still offer the possibility of fertilization from donors’ eggs in medical institutions outside Israel (e.g. from Cyprus, Ukraine, and Romania). According to a media report in the television’s weekend magazine (February 27th 2004), couples travel to places where eggs are retrieved from a paid donor, and are transplanted into the womb of the recipient. After this legislative change, the future of cross-border reproductive care for egg donation depends on the development of supply.

Legal restrictions on surrogacy for same sex couples and single parents led numerous couples to consider surrogacy outside of Israel (e.g., India). With no known intention to change this restriction, it is unlikely that travelling abroad for surrogacy, at least for same sex couples, will decrease or stop in the near future.

In conclusion, in Germany assisted reproductive treatment is regulated by restrictive legislation based on moral concerns, while in Israel there is a more liberal attitude toward options of reproductive technology. However, in both countries homosexual couples and single parents do not have access to all assisted reproductive treatments available. Thus, in Israel and Germany, policies create a demand for egg donation and surrogacy abroad and have engendered public debate about “reproductive tourism”.

## Methods

To identify different use of the term “tourism”, we conducted an internet-based pre-search by keywords. We then analyzed identified articles to determine the precise terms used to describe the practice and phenomenon of travelling patients^d^. In order to define how the public medica discourse in Germany and Israel make use of the term “tourism”, different contexts and connotations were analyzed through the following questions:

1. Are the different terms used in distinct contexts?

2. What characteristics of the practice and phenomenon are considered when the term “tourism” is used in German and Israeli public media?

3. What characteristics are considered when the term “tourism” is avoided?

We expected that cultural connotations would be easier to detect when concentrating on discourses in the respective native languages. We therefore focused on websites such as I-forums, online newspapers, articles, etc. in both countries, and in Germany focused upon printed media as well. Through the analysis we identified particular cultural, legal and social perceptions of the use of the term “tourism”. We compared the results from Israel and Germany and suggested reasons for identified differences.

## Results and analysis

In our analysis of the public media discourse we will discern different uses of the term “reproductive tourism”. We will, in particular, focus on the metaphorical use of the term. Metaphors are a linguistic instrument to implicitly express values as well as facts. The public media discourse reflects and also influences public opinion; it thereby can have a normative function. Consequently, analysis of the different uses of the term can show how a situation or phenomenon is described and evaluated in a society. We herein present the main findings regarding the contexts and references of the analyzed terms; we classified the sources, background conditions, and target population of the references, as well as the different contexts according to salient common characteristics.

### The public media discourse in Israel

In Israel, we searched the internet in Hebrew for different references that use any term related to tourism in the field of reproductive treatment. About 30 items were found, not all of which were relevant. In all, 16 relevant references were identified and analyzed. The characteristics were considered according to the context and the different use of the term. The Israeli public is very interested in reproductive technologies. Search results from the internet clearly showed that all of the popular newspapers deal with subjects relating to cross-border reproductive care. It should be mentioned that articles also used other words when reviewing the phenomenon. The subject was also raised in the parliament; therefore two references were published by the ministry of health and the parliament’s research centre. Other references were retrieved from informative/publication websites of clinics, supplying information regarding their services and the process to potential clients, or from patients forums.

In Israel, the terms “fertility tourism” or “reproductive tourism” () are used to imply the consequence of regulation differences between countries and in order to lobby for a change in legislation that would make these “services” legally accessible [24]. The language reflects a market model referring to a “deficit” in eggs: “The race after the eggs: the rising demand in IVF brought on a flourishing global market of eggs” [25]. Yet, “egg tourism” was also used in the Knesset, when discussing the new egg donation bill as something not wanted in Israel [26].

When the right to parenthood is the focus of the article, a more neutral term is used (“cross-border reproductive care”) [27]. When patients in Israel are directly addressed, the term “tourism” is never used (), but instead is referred to as “to purchase eggs” (), “to import” () or simply “egg donation abroad” () [16,28-30]. However, we have found patients using the term “tourism” in I-forums, for example, when talking about a “travel agent” that “specializes” in “fertility tourism”. The forums are often used by infertile people seeking information about an opportunity to have a child. Other prospected parents receiving information about the procedure in a surrogacy centre stated: “…it runs like *a business, a baby market*, they talk with us about the *costs of a womb for rent*” [31].

The expression “womb for rent” () was used in several contexts: First, by feminist organizations that objected to the law that enables surrogacy in Israel: “the law encourages treating women as an instrument to deliver babies – a womb for rent” [32]. Second, by an infertile woman who described that she wanted minimal social contact with the surrogate mother: “I only wanted a womb for rent” [33]. Third, when describing negative consequences of poverty: “The solution to economic agony: a womb for rent” [34]. Another expression related to market vocabulary was “surrogacy industry” (); this was used in an article about surrogacy in India to express human rights activists’ objections. Another article dealing with the “international fertility market” in India also used the expression “surrogacy industry” [35]. Another example of market vocabulary is the expression “to buy eggs” () that was cynically used in Israel in order to describe senior gynecologists offering infertile women the opportunity “to buy” eggs abroad, while choosing the “donor” according to her physical traits [36].

In summer 2009, the state of Romania charged some Israeli doctors with trafficking human eggs. The doctors arrived in Romania with their Israeli patients in order to recruit eggs from Romanian women. Most media reports dealing with this affair used terms like “egg trafficking” [37], or “egg sale” [38]. Since the incidence in Romania, a new legal vocabulary that implies trade and trafficking, was developed to express concerns regarding the ramifications of such practices.

It is interesting that the terminology in Israel appears to be quite functional. Different terms are used for various target populations, and for different purposes: when recruiting potential clients (or customers), and/or when addressing infertile couples, it is customary not to use the term “tourism”. Rather, a more neutral, descriptive term will be used, such as “IVF patient/service”. Moreover, when addressing these populations, the term “donation” is used to describe the supply of reproductive services despite the fact that the intention is to buy and sell these services. Nevertheless, the term “tourism”, or even terms such as “trafficking” that may have a more negative connotation, are used when discussing the economic aspects of such practices, or as legal accusations against doctors.

### The public media discourse in Germany

In Germany, we reviewed five of the most important and opinion-leading newspapers and magazines: “Der Spiegel”, “Die Zeit”, “die tageszeitung”, “Frankfurter Rundschau”, “Frankfurter Allgemeine Zeitung (FAZ)”^e^.

Altogether 63 articles published in the years 2000 - 2010 were found, 35 used a tourism metaphor or treated the topics of egg donation or surrogacy in the context of travelling for reproductive health care; 18 out of the 35 articles analyzed used a term that contains the word “tourism” in the context of medical treatment. Three (3) articles of the 18 used a term related to “tourism” generically to indicate the phenomenon. Thirteen (13) articles out of 18 mainly dealt with the commercial aspects of the phenomenon. The FAZ article “500.000 eingefrorene Embryos”(“500.000 frozen embryos”) states: “The business of IVF, sperm banks, egg cell donation and surrogate mothers has become one of the fastest growing industries of South California [39]. “Only 9 of 35 articles mentioned the donors’ point of view. Of these 9 articles only one, reporting on the surrogate mother and/or egg donators, used a term related to “tourism”, the other 8 did not make any reference to the term. Six (6) of these articles dealt with commercial aspects of surrogacy, but included the perspective of the infertile couples. For example, the article “Verbotene Kinder” (“Forbidden Children”) appearing in “Die Zeit” elaborates in detail on the stresses of the infertile couple as well as the surrogate mothers’ situation [40]. In the article, vocabulary from the market is used to describe the economic aspects, yet the term “tourism” is avoided. In the whole sample, the physicians’ point of view is mentioned more often than the donors’ perspective: Twelve (12) of the articles report on physicians’ perspective, six (6) of them using the term “tourism”.

In the public media discourse in Germany, the terms “fertility tourism” or “reproductive tourism” are most commonly used in a commercial context, for example, by calling/denouncing the practice of travelling for reproductive purposes an industry, a business, or an opportunity for making money. The German magazine “Der Spiegel”, for example, writes: “Those to whom the *supply* of the German baby mills is not sufficient, turn to foreign countries because the test-tube procreation *market* has become a global one for a long time” [41]. Another “Der Spiegel” article, “Babies made to order” states: “A child at your request? No problem in the USA: There are companies finding surrogate mothers, egg or sperm donation in California. The business booms – also with clients from overseas” [20]. The German newspaper “Die Zeit” states: “Nowhere else in the world is it allowed to do *business* with sperm and egg cells according to the *rules of supply and demand*” [42].

The term “tourism” in the German public debate emphasizes the business aspect of the phenomenon. When the physicians’ point of view is reflected, the use of the metaphor is ambivalent. When it is avoided, the article usually discusses the phenomenon in a broader perspective such as that of the person travelling abroad for these services and/or the one of the surrogate/egg donor. We find that the use of the term "tourism" is not a coincidence. It is interesting that both in Israel and Germany, the term was not used to express the fact that the travelling is done for a specific purpose, for pleasure, or cultural enjoyment. Rather, it is used to illustrate, and to criticize the economic aspects of these services and practices.

### Tourism and reproductive treatment

One can identify two main contexts, in which the term tourism is usually understood and interpreted: First, the context of leisure and fun, and second, the industrial and economic context. According to common understanding, “tourism” refers to travel to another country for a vacation, in order to enjoy its natural and/or cultural resources.

But the term “tourism” is also an economic term. The Oxford Dictionary defines tourism as “the commercial organization and operation of holidays” [43]. Tourism as part of the service industry is of high significance for global economics.

The World Tourism Organization defines tourists as people who “travel to and stay in places outside their usual environment for more than twenty-four (24) hours and not more than one consecutive year for leisure, business and other purposes not related to the exercise of an activity remunerated from within the place visited” [44].

This definition does not limit the purpose of tourism, and we think that this definition can easily fit “reproductive tourism” in three dimensions. First, it is well known that there are many specific market segments that use tourism for “other purposes”, like sports tourism, music tourism, business tourism etc. Receiving reproductive health care can be seen as such a purpose. Describing travellers as “fertility tourists” also matches the concept of tourists travelling for business purposes or in search of a cure. Second, “the purpose of leisure” can express the leisure of having a family or enjoying the idea of having a child. Finally, even the classical motive “to enjoy a country's culture” can apply to medical tourists. They benefit from a country’s more liberal attitude(s) towards reproductive treatment, and possibly from economic circumstances that motivate women to supply reproductive services to anyone who can afford to pay.

### Ethical analysis

Although the phenomenon of patients travelling for reproductive treatment can fit into definitions of “tourism”, some ethicists have raised concerns regarding the implicit normative connotations of the term. Ritu Priya, at the Centre for Social Medicine and Community Health at Jawaharlal Nehru University in New Delhi, remarks that using the notion “health tourism is likely to distort the perspectives of health providers, promoting medicine as a purely commercial venture” [45]. The legal scholar Richard Storrow also criticizes the use of the term by providers; he argues that marketing the reproductive treatment industry implies that the journey is not only framed by the “fantasy” of conceiving a child, but also to do so during romantic holidays. An exotic vacation is promised, during which the medical treatment is accompanied by entertainment and pleasure, instead of staying in a hospital and receiving medical treatment with all its potential side-effects burdens and risks. This concept is presented as analogous to planning a vacation with the help of a travel agent, rather than scheduling a medical procedure [46]. By using the term “tourism” that, according to Storrow, is mainly understood “as a type of travelling that involves leisure, pleasure and free time”, the providers may conceal the realities of the journey, i.e., the medical treatment.

Yet, given that infertile couples often describe their condition as devastating, and their effort to conceive a child as stressful and requiring enormous physical and emotional strength, it seems difficult to harmonize the realities of “reproductive tourism” with the idea of tourism as pleasure travel [5]. In her empirical research with couples that travelled to other countries for reproductive treatment, Marcia Inhorn discovered that, for the patients, the use of the term seems insensitive given the stressful and expensive journey they had to undertake. Most of the patients would prefer legal, trustworthy, and economical services available closer to their home(s), having the impression that “…’reproductive tourism’ sounds like a ‘gimmick’, which does not take infertile people’s suffering seriously” [47].

Therefore, different terms have been suggested. Roberto Matorras, president of the “Sociedad Espanola de fertilidad”, argues that the term “tourism” may be offensive both to couples and to professionals involved in the process [48]. In order to focus on the patients, Matorras suggests the term “reproductive exile”. In his opinion, this better expresses the difficulties and constraints faced by infertile patients who are forced to travel globally for assisted reproduction.

Guido Pennings agrees with Matorras that “tourism” is a derogatory term when used in the medical context. The term, he argues, suggests that the desire of the patients is insignificant, or that they are looking for something exceptional or “bizarre”, although most patients simply want to avoid long waiting lists or high costs. Pennings, however, holds that the term “exile” creates the misleading impression that patients are forced to go abroad as a punishment. In a reply to Matorras, he suggests replacing the term “tourism” by a more neutral term, such as “cross border reproductive care”, which does not carry the problematic connotations of “tourism”, and its accompanying value judgments [49]. Contrary to this criticism, Kristen Smith states that the term “tourism” in a medical context *can* be used in a constructive way, as “…the bioethical dimensions of the practice and industry are brought into a much sharper focus than could be achieved with a more benign descriptor such as the term ‘medical travel’” [50].

Our findings show that in both Germany and Israel, the use of the metaphor “reproductive tourism” and its hidden messages depend on the writers’ intention and the target population. We can differentiate two opposite positions: the one of the critics, and the one of the providers. While the critics’ intention in using the term is to illustrate their criticism by using the metaphor “tourism” as an economic term, providers use it to justify their business on one hand, and to trivialize the efforts of their potential patients on the other. Providers and the public media express different values of the phenomenon by using the same term. Both can make use of the term according to agendas that either condemn or trivialize the underlying social phenomenon.

## Conclusions

In the public debate the term “reproductive tourism” is used to highlight certain aspects of seeking medical treatment to conceive or bear a child. Metaphors are common linguistic instruments illustrate a particular position. But metaphors usually do not reflect all aspects of the phenomenon. “Reproductive tourism” does not reflect patients’ reality, in particular the stress and exertion involved. Patients therefore feel offended by the term. Thus, some ethicists suggest using different metaphors for the practice, and its various aspects and actors.

Terms like “tourism” or “exile” tend to be biased in that they have represent value judgements, that implicitly stress a certain aspect or meaning. In an ethical debate, however, positions and arguments should be explicitly and clearly stated. Implicit criticisms through the use of metaphors lead to misunderstandings, imprecision and inappropriate generalization. Therefore, ethicists should be cautious when using these metaphors that are adapted from public discourse. Our findings support Guido Penning’s suggestion to use unloaded term, such as cross-border reproductive care to denominate the practice in a more neutral way, and to ensure that its meanings is explicit, when engaging in debate and/or criticism.

## Endnotes

^a^All translations are made by the authors.

^b^Orthodox Jews consider it to be a religious duty to “be fruitful and multiply”. The curse of female barrenness is a consistent and profound theme in the bible and in Jewish tradition. The words of Rachel to Jacob “give me children or else I die” (Genesis 30:1) continue to reverberate in Israeli culture. According to *halakha* (Jewish law), which governs the laws of marriage and divorce for Jews in Israel, a man has the right to divorce his wife if the marriage has failed to produce children over ten years.

^c^Paragraph 6(e) of the Second Addendum to the National Health Insurance Law, 1994, *S.H.* no. 1469, p. 183.

^d^Four terms that use the tourism metaphor were found: “Fortpflanzungstourismus” (“reproduction tourism”), “Fertilitätstourismus” (“fertility tourism”), “Reproduktionstourismus” (“reproduction tourism”) and “Fruchtbarkeitstourismus” (“fecundity tourism”), in Hebrew: , , , . We searched all articles that use one of these “tourism” terms. Time period for our search was in both contexts 10 years: (Jan.01st, 2000 until Jun.19th, 2010).

We also used other keywords “Leihmutter” (“surrogate mother”),  and “Eizellspende” (“egg donation”),  linked to the words “Ausland” (“foreign country”),  or “Reise” (“travel”),  in order to find all articles dealing with our research topic.

^e^“Der Spiegel” is known for its investigative journalism, “die tageszeitung” for its left-wing tendencies. “Die Zeit” is an independent weekly newspaper that is widely recognized as liberal. While the “Frankfurter Rundschau” is considered as social liberal the “Frankfurter Allgemeine Zeitung” is known as classical liberal with conservative tendencies.

## Competing interests

The authors declare that they have no competing interests.

## Authors’ contributions

SB participated in the design of the study, performed the analysis especially for the parts of the article that concern the situation in Israel and drafted the manuscript. MAM participated in the design of the study, performed the analysis especially for the parts of the article that concern the situation in Germany and contributed in drafting the manuscript. All authors read and approved the final manuscript.
